# Effects of 12-month probiotic supplementation on bone mineral density and bone turnover markers in early postmenopausal females: A double-blind randomized controlled trial

**DOI:** 10.1016/j.ajcnut.2026.101384

**Published:** 2026-06-08

**Authors:** Stephanie Resciniti, Pauline Dacaya, Kanny Yim, Tammy Esmaili, Antony Vinh, Hericka BF Galvão, Ali Ghasem-Zadeh, Jessica R Biesiekierski, George Moschonis

**Affiliations:** 1 Department of Sport, Exercise and Nutrition Sciences, Discipline of Food, Nutrition, and Dietetics, School of Allied Health, Human Services and Sport, La Trobe University, Melbourne, VIC, Australia; 2 Faculty of Medicine, Dentistry and Health Sciences, The University of Melbourne, Melbourne, VIC, Australia; 3 Centre for Cardiovascular Biology and Disease Research (CCBDR), La Trobe Institute of Medical Science (LIMS), La Trobe University, Melbourne, VIC, Australia; 4 Department of Microbiology, Anatomy, Physiology and Pharmacology, School of Agriculture, Biomedicine and Environment, La Trobe University, Melbourne, VIC, Australia; 5 Department of Medicine and Endocrinology, Austin Health, The University of Melbourne, Heidelberg West, VIC, Australia; 6 Human Nutrition Group, School of Agriculture, Food and Ecosystem Sciences, The University of Melbourne, Melbourne, VIC, Australia; 7 European Centre for Obesity, Harokopio University, Athens, Greece; 8 Department of Nutrition and Dietetics, School of Health Science and Education, Harokopio University, Athens, Greece

**Keywords:** probiotics, supplementation, bone mineral density, bone turnover markers, postmenopausal females, randomized controlled trial

## Abstract

**Background:**

Early postmenopausal females experience rapid bone loss, and modulation of the gut–bone axis has been proposed as a preventive strategy. However, the role of probiotics relative to established lifestyle factors such as nutrition and physical activity remains unclear.

**Objectives:**

To evaluate whether 12 mo of supplementation with a lactobacilli-based probiotic attenuates bone loss in early postmenopausal females.

**Methods:**

In this double-blind, randomized, placebo-controlled trial, 114 females 1 to 8 y postmenopause were allocated to a daily probiotic (*Lactiplantibacillus plantarum* DSM15312, DSM15313, and *Lacticaseibacillus paracasei* DSM13434) or placebo. The primary outcome was change in distal tibia total volumetric bone mineral density (BMD) assessed by high-resolution peripheral quantitative computed tomography. Secondary outcomes included additional high-resolution peripheral quantitative computed tomography parameters, areal BMD by dual-energy X-ray absorptiometry, and circulating biomarkers. Intention-to-treat analyses were conducted using linear mixed-effects models, adjusted for baseline covariates; secondary outcomes were corrected for multiple comparisons.

**Results:**

A total of 114 participants were randomly assigned, and all available data were included in the analyses. Baseline characteristics were broadly comparable. Over 12 mo, both groups experienced modest declines in bone density. The primary analysis showed a modest but statistically significant greater decline in distal tibia total volumetric BMD in the probiotic group compared with placebo (−3.7 mg HA/cm^3^; 95% confidence interval: −6.9, −0.4 mg HA/cm^3^; *P* = 0.027). Similar trends were observed at other peripheral sites, but most secondary outcomes were not significant after multiple testing correction. No treatment effects were observed for bone turnover, calciotropic, or inflammatory markers.

**Conclusions:**

In early postmenopausal females, 12-mo supplementation with a lactobacilli-based probiotic does not attenuate bone loss and was associated with a small but statistically significant greater reduction at the primary site. These findings do not support this probiotic formulation as a standalone intervention for skeletal preservation.

This trial was registered at the Australian and New Zealand Clinical Trials Registry as ACTRN12621000810819.

## Introduction

Osteoporosis is a systemic skeletal disorder characterized by reduced bone mass and deterioration of bone microarchitecture, increasing susceptibility to fragility fractures. Affecting over one-third of females over 50 [1], fragility fractures cause profound morbidity and mortality, with annual costs projected to exceed €56.9 billion in Europe [2] and AU$67.9 billion over the next decade in Australia [3]. Crucially, over half of fragility fractures occur in females outside the osteoporotic diagnostic threshold (T-score ≤−2.5) [4], highlighting the urgent need for preventive strategies before irreversible skeletal loss occurs.

The initial 5 to 6 y post menopause represents a critical preventive window, as estrogen deficiency triggers accelerated bone loss of 2% to 2.5% annually [[5], [6], [7], [8]]. This decline promotes osteoclastogenesis and increases bone resorption, compromising trabecular-rich sites [[9], [10]]. Although bone mineral density (BMD) is the clinical assessment standard, shifts in bone turnover markers (BTMs), such as procollagen type I N-terminal propeptide (PINP), osteocalcin (OC), and C-terminal telopeptide (CTX), may detect earlier remodeling shifts [11]. Monitoring BTMs alongside areal and volumetric BMD (aBMD and vBMD) offers a more comprehensive assessment of bone-protective interventions.

Beyond hormonal influences, osteoporosis is driven by nutrient deficiencies, including inadequate calcium and vitamin D intake [12]. Vitamin K_2_ also plays a critical role in bone metabolism through OC γ-carboxylation, a process essential for mineralization [[13], [14], [15], [16], [17], [18]]. The gut microbiome further supports skeletal health by regulating immune responses, systemic inflammation, intestinal barrier integrity, endocrine signaling, pathogenic overgrowth, and vitamin biosynthesis [19,20]. Consequently, menopausal dysbiosis may accelerate bone loss by increasing intestinal permeability and chronic low-grade inflammation while impairing calcium absorption and bone metabolism [19,20].

Preclinical evidence strongly supports the role of gut microbiota modulation in skeletal health. In ovariectomized rodents, *Lactobacillus* strains prevented bone loss and improved bone microarchitecture [[21], [22], [23]]. Probiotics appear to suppress pro-inflammatory cytokines (e.g., TNF-α, IL-6), reduce receptor activator of nuclear factor κ-B ligand (RANKL)-mediated osteoclast activation [24,25] and enhance vitamin K_2_ synthesis. However, results from 9 clinical trials in postmenopausal females remain inconsistent [[26], [27], [28], [29], [30], [31], [32], [33], [34]] with some reporting modest single-site improvements. Heterogeneity was likely attributed to inadequately powered designs, variability in strains, durations, sample sizes, age, years since menopause, and endpoints assessed [35,36]. Six studies examined probiotics in isolation [26,[30], [31], [32], [33], [34]] and only 2 utilized advanced imaging techniques such as high-resolution peripheral quantitative computed tomography (HR-pQCT) [27,30].

This trial aims to address these gaps by assessing the impact of 12 mo daily probiotic supplementation (*Lactiplantibacillus plantarum* DSM15312, *L*. *plantarum* DSM15313, and *Lacticaseibacillus paracasei* DSM13434) on bone health in females within 8 y of menopause. This lactobacilli combination has demonstrated bone-protective effects in ovariectomized animal models [22] and attenuated lumbar spine bone loss in early postmenopausal females [34] potentially via modulating inflammatory pathways and gut–bone signaling [37]. We hypothesize that probiotic supplementation will mitigate bone loss relative to placebo, mediated by beneficial gut microbiota shifts, reduced systemic inflammation, and improved bone mineralization.

## Methods

### Study design

The Probone21 study was a 12-mo, double-blind, randomized, placebo-controlled trial in healthy early postmenopausal females, with measurements taking place at La Trobe University (Bundoora, Victoria, Australia) and the Austin Repatriation Hospital (Heidelberg, Victoria, Australia). Participants were randomly assigned in a 1:1 ratio to receive either a probiotic supplement or placebo. The trial adhered to the Standard Protocol Items: Recommendations for Interventional Trials (SPIRIT) guidelines, received approval from the La Trobe University Human Research Ethics Committee (HREC 21038), and was registered with the Australian and New Zealand Clinical Trials Registry (ACTRN12621000810819). A detailed description of the trial design is available in the published study protocol [38].

### Study Participants

Eligible females were aged 45 to 65 y, nonsmokers, between 1 y and 8 y post menopause, with a BMI (in kg/m^2^) of 18 to 32 and stable weight for ≥6 mo. Exclusion criteria included diagnosed bone disease, systemic conditions known to affect bone or inflammation (e.g., hypothyroidism, rheumatoid arthritis, inflammatory bowel disease), use of glucocorticoids or hormone replacement therapy within the prior 12 mo, history of malignancy in the past 5 y, or excessive alcohol (>4 drinks per day) or caffeine (>5 cups per day) intake. If participants violated the aforementioned restrictions, they were excused from further participation in the study. All participants underwent screening dual-energy X-ray absorptiometry (DXA), with only those with a T-score >−2.5 at the lumbar spine or total hip eligible for enrolment. Females with T-scores ≤−2.5 were referred to their healthcare provider. Recruitment was achieved via professional networks, university mailing lists, and targeted social media advertising from December 2021 to June 2023. Amendments to eligibility criteria during recruitment increased the upper thresholds for years since menopause (6–8) and BMI (30–32), to enhance enrolment feasibility without compromising validity. Written informed consent was obtained from all participants.

### Randomization and Blinding

A computer-generated block randomization sequence (block size = 10) was prepared by an independent researcher. Allocation was communicated only to the manufacturer, who packaged probiotic and placebo products in indistinguishable, sequentially numbered containers. Investigators, staff, and participants were blinded to allocation until after database lock and final analysis. Further information on allocation procedures is detailed in the trial protocol [38].

### Intervention

The investigational product contained a blend of *L*. *plantarum* DSM15312, *L. plantarum* DSM 15313, and *Lacticaseibacillus paracasei* DSM13434 (herein referred to as lactobacilli combination), provided as freeze-dried powder in capsules [1 × 10^10^ colony-forming unit (CFU) per capsule]. As specified by the manufacturer, the study product had a 3-y shelf life under standard ambient storage conditions. Product stability was monitored by assessing the viability of the probiotic bacteria at 0 mo, 6 mo, 12 mo, 18 mo, and 24 mo using a CFU counting method in accordance with standard laboratory procedures [Nordic-Baltic Committee on Food Analysis (NMKL), NMKL Procedure number 23: Guide on quality assurance in microbiological laboratories (NMKL, Oslo/Bergen)]. Participants consumed 1 capsule daily with water, with or without food, for 12 mo. Placebo capsules were identical in appearance and taste but contained maize starch. Adherence was monitored through capsule counts at follow-up visits.

### Restrictions during the study

Participants were instructed to refrain from consuming other probiotic products, calcium or vitamin D supplements, or initiating hormonal therapies. They were required to maintain body weight within ±5 kg of baseline, avoid alcohol within 24 h of visits, fast overnight (water permitted), and abstain from strenuous physical activity within 24 h prior to assessments. Lifestyle guidance emphasized maintaining habitual physical activity and diet throughout the study period.

### Outcomes and assessments

#### Socio-demographic and anthropometric data collection

At baseline, participants completed a standardized socio-demographic questionnaire administered electronically via Research Electronic Data Capture (REDCap) software to collect information on age, education, occupation, employment status, marital status, ethnicity, country of birth, and residential area. Medical history and concomitant medication use were also obtained to record potential confounders. Anthropometric assessments were conducted by trained staff at each study visit. Body weight was measured to the nearest 0.1 kg using a calibrated digital scale (Seca), whereas standing height was recorded to the nearest 0.5 cm using a stadiometer (Leicester height measure; CMS Instruments). All measurements were performed with participants wearing light clothing and no shoes. These data were used to calculate BMI, which served both as a descriptive characteristic and as an adjustment covariate in analyses.

#### Dietary intake

Dietary intake was assessed at each time point using 3-d food diaries and analyzed with FoodWorks (Xyris Software), a common nutrition analysis program in Australia. Dietary energy, macronutrient, and micronutrient intake values were derived from the analysis of these food diaries. Participants were instructed to record all food and beverage consumption over 2 typical weekdays and 1 weekend day, representative of their usual dietary habits.

#### Physical activity

9The Active Australia questionnaire, a validated tool for assessing physical activity [39] in Australian middle-aged females, was used to collect data at baseline and 12 mo. Participants reported the frequency and duration of time spent in the past week walking briskly, engaging in moderate-intensity leisure activities, and participating in vigorous-intensity physical activity.

#### BMD measurements

The primary outcome was change in distal tibia total vBMD measured by HR-pQCT (XtremeCT, Scanco Medical). This technique also provided detailed measures of bone microarchitecture, including total vBMD, cortical thickness, cortical vBMD, and trabecular bone volume fraction at both tibia and radius. All scans were performed at baseline and 12 mo using standardized positioning with anatomically contoured carbon fiber casts. Regions of interest were determined using a fixed offset method, with reference lines set at 9.5 mm and 22.5 mm proximal to the distal radius and tibial plafond, respectively. Precision was high, with coefficients of variation (COV) of 0.61% for the tibia total vBMD and 0.96% for radius total vBMD in reproducibility testing.

Secondary bone outcomes included changes in aBMD at the lumbar spine (L1–L4), total hip, femoral neck, and forearm, assessed with DXA (Hologic Inc.). The DXA device was calibrated daily using a phantom, and all scans were analyzed by a single trained operator to reduce variability. A precision sub-study of 45 females from this cohort demonstrated COV of 1.5% for lumbar spine, 1.2% for total hip, 1.5% for femoral neck, and 1.6% for forearm.

#### Body composition measurements

Body composition was evaluated using both bioelectrical impedance analysis and DXA to provide complementary measures of fat mass and lean body mass. Bioelectrical impedance analysis was conducted at each time point using the Tanita MC-780 multi-frequency segmental body composition analyzer (Tanita Corporation), with participants in a fasted state and assessed under standardized conditions. DXA scans (Hologic Inc.) were also performed by a trained researcher at baseline and 12 mo to assess whole-body and regional composition. All measurements were conducted in accordance with the manufacturer’s standard protocols.

#### Blood biomarker measurements

Venous blood samples were collected at baseline and 12 mo following an overnight fast. Samples were processed within 2 h, divided into aliquots, and stored at −80°C until batch analysis. All assays were performed using commercially available kits according to manufacturer instructions. Details for each assay, including type, manufacturer, COV, and limits of detection, are provided in Supplementary Table 1. Inter- and intra-assay COV were within acceptable ranges. A broad panel of biomarkers relevant to bone metabolism, inflammation, and nutrient regulation was assessed.

Bone formation markers included PINP, measured by electrochemiluminescence immunoassay with high sensitivity and reproducibility, and total OC, quantified using chemiluminescent immunoassay. Bone resorption was evaluated by the β-isomer of the CTX of type I collagen and tartrate-resistant acid phosphatase isoform 5b, both widely validated indices of collagen degradation and osteoclast activity. In addition, regulatory markers of osteoclast differentiation were assessed, including osteoprotegerin and soluble RANKL, providing insight into the balance of bone resorption signaling pathways.

Calciotropic hormones were measured comprehensively: intact parathyroid hormone, circulating 25-hydroxyvitamin D_2_/D_3_ [25(OH)D_2_/D_3_] via liquid chromatography–tandem mass spectrometry, active 1,25-dihydroxyvitamin D, and calcitonin. Vitamin K status was examined indirectly through undercarboxylated OC, a sensitive marker of γ-carboxylation.

Systemic inflammation was captured by high-sensitivity C-reactive protein (hs-CRP), IL-6, and TNF-α, whereas renal and hepatic function were monitored using creatinine, urea, aspartate aminotransferase, and alanine aminotransferase assays.

All procedures were performed at accredited pathology laboratories and at the La Trobe Nutrition Clinical Trial Laboratory, ensuring methodological rigor and reproducibility [38].

#### Sample size

Based on prior HR-pQCT studies, a between-group difference of 1% in tibial vBMD (SD 1.78%) was assumed [30]. To achieve 80% power at α = 0.05, 50 females per arm were required. Allowing for 20% attrition, the target sample size was 124.

### Statistical analysis

All statistical analyses were conducted using SPSS (version 31.0; IBM Corporation). Continuous variables are presented as mean ± SD, and categorical variables as counts (percentages). Baseline characteristics were summarized descriptively without formal statistical comparison between groups, in line with recommendations for randomized trials. Clinically relevant variables were included as covariates in adjusted analyses.

The primary outcome was change in distal tibia total vBMD. Secondary outcomes included other HR-pQCT-derived bone parameters, DXA-derived aBMD, circulating biomarkers, and dietary intake variables.

Changes over time were analyzed using linear mixed-effects models with participant-specific random intercepts to account for within-subject correlation between baseline and 12-mo measurements. Fixed effects included treatment group (probiotic compared with placebo), time (baseline and 12 mo), and the group × time interaction. The primary treatment effect was defined as the group × time interaction, representing the adjusted between-group difference in change over 12 mo.

All models were adjusted for pre-specified baseline covariates, including years since menopause, BMI, dietary calcium intake, serum 25(OH)D, and moderate and vigorous physical activity. When serum 25(OH)D concentrations or dietary calcium intakes were the dependent variables, these were not included as covariates in the relevant models. Model assumptions were assessed using residual plots and Q–Q plots. Linear mixed models were estimated using restricted maximum-likelihood and included all available observations, consistent with a modified intention-to-treat approach under the missing-at-random assumption. The primary outcome was not adjusted for multiple comparisons. For secondary outcomes, *P* values for the group × time interaction was adjusted within outcome domains using the Benjamini–Hochberg false discovery rate (FDR) procedure. Results are presented as adjusted mean changes with 95% confidence intervals (CIs) and corresponding *P* values. Statistical significance was defined as a 2-sided *P* < 0.05.

## Results

**FIGURE 1:**
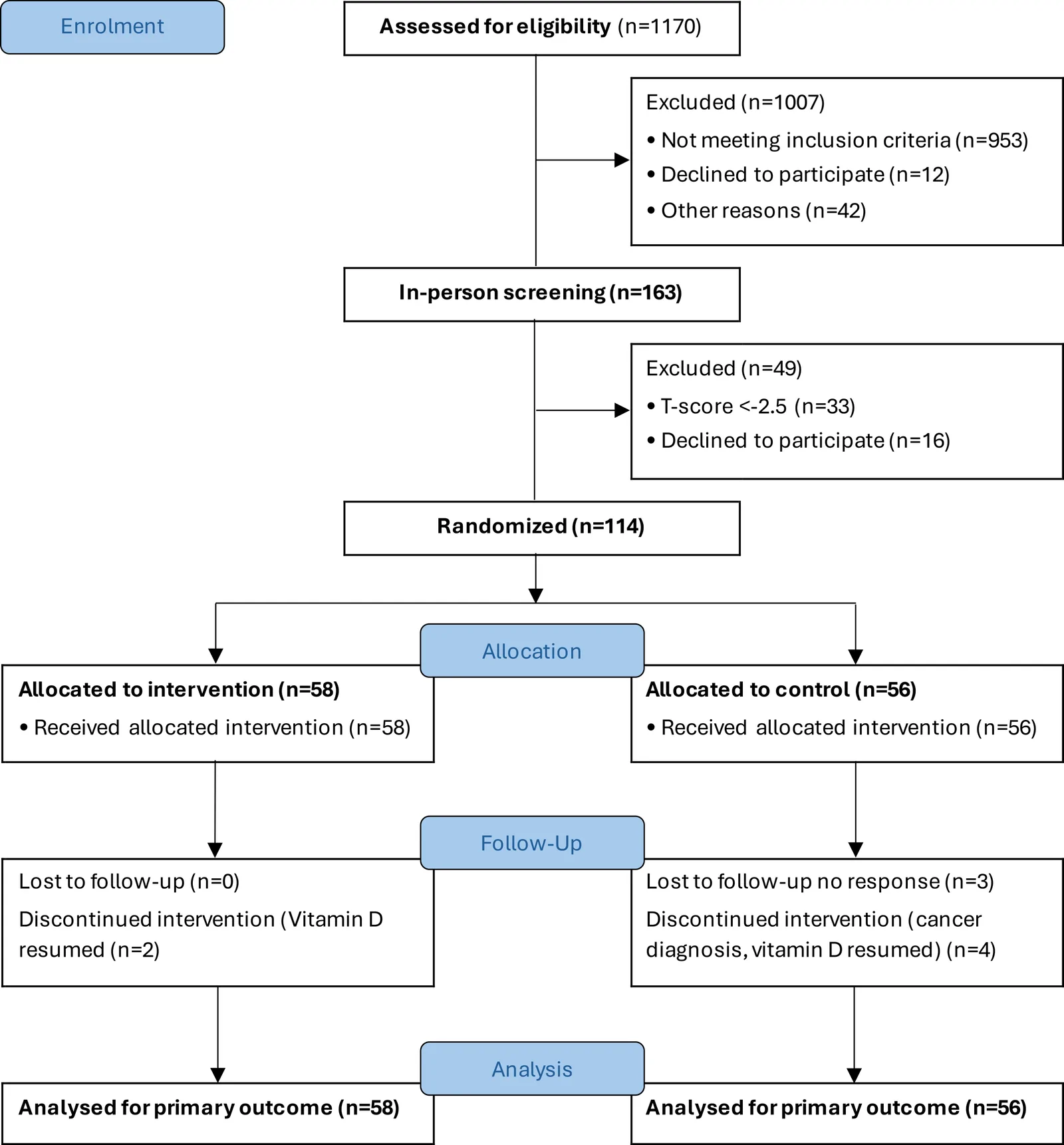
Flow diagram of study participants across study time points.

The CONSORT diagram in Figure 1 outlines the flow of volunteers and participants across the different time points of the study.

### Participant characteristics

A total of 114 postmenopausal females were randomly assigned (58 probiotic; 56 placebo). Baseline characteristics were broadly comparable between groups (Table 1). Participants had a mean age of ∼56 y and were, on approximately, 4 y postmenopause. Anthropometric measures, bone density, and most dietary and lifestyle variables were similar across groups. The placebo group reported lower dietary calcium intake and higher walking time than the probiotic group.

**TABLE 1:** Baseline characteristics of study participants by treatment group

	Probiotic (*n* = 58)	Placebo (*n* = 56)
Demographics and anthropometrics, mean (SD)		
Age (y)	56.5 (4.1)	56.2 (3.8)
Years since menopause	3.9 (1.7)	3.9 (1.7)
Weight (kg)	67.7 (10.6)	67.2 (12.0)
Height (cm)	163.5 (6.1)	164.8 (6.1)
BMI (kg/m^2^)	25.3 (3.4)	24.2 (5.1)
BMI categories, *n* (%)		
Underweight	1 (1.7)	2 (3.6)
Normal weight	25 (43.1)	28 (50.0)
Overweight	28 (48.3)	22 (39.3)
Obese	4 (6.9)	4 (7.1)
Ethnicity, *n* (%)		
Caucasian	55 (94.9)	54 (96.4)
Asian/Indian	2 (3.4)	2 (3.6)
Indigenous Australian	1 (1.7)	0 (0)
Work status, *n* (%)		
Full time	30 (51.7)	22 (39.3)
Part time	15 (25.9)	23 (41.1)
Casual	4 (6.9)	6 (10.7)
Retired	6 (10.3)	5 (8.9)
Unemployed	3 (5.2)	0 (0)
Current medication, *n* (%)		
Yes	13 (22.4)	20 (35.7)
No	45 (77.6)	36 (64.3)
Current supplement use, *n* (%)		
Yes	28 (48.3)	32 (57.1)
No	30 (51.7)	24 (42.9)
Bone density (lumbar spine) (g/cm^2^)	0.923 (0.107)	0.923 (0.129)
T-score score	‒1.119 (0.942)	‒1.075 (1.155)
T-score category, *n* (%)		
Normal (≥‒1)	23 (39.7)	22 (39.3)
Osteopenia (‒2.5, ‒1)	35 (60.3)	34 (60.7)
Dietary intake, mean (SD)		
Total energy (kJ/d)	8195.4 (2,791)	8673.8 (1,973)
Protein (g/d)	84.1 (30.2)	91.9 (23.6)
Protein (g/kg/d)	1.3 (0.5)	1.4 (0.4)
Carbohydrates (g/d)	177.2 (88.4)	188.4 (57.2)
Total fat (g/d)	84 (30.2)	94.4 (31.7)
Fiber (g/d)	26.9 (11.2)	27.9 (10.4)
Alcohol (g/d)	11.1 (13.8)	8.8 (11.8)
Calcium (mg/d)	856 (436)	1003 (356)
Phosphorus (mg/d)	1460 (583)	1566 (385)
Magnesium (mg/d)	369 (127)	398 (124)
Sodium (mg/d)	2274 (932)	2407 (813)
Potassium (mg/d)	3452 (1557)	3478 (853)
Zinc (mg/d)	9.8 (3.4)	10.4 (2.5)
Vitamin C (mg/d)	110.0 (70.4)	109.7 (63.4)
Physical activity, mean (SD)		
Walking (min/wk)	199.7 (187)	320.5 (123)
Moderate activity (min/wk)	74.6 (123)	109 (172.5)
Vigorous activity (min/wk)	67.3 (89.3)	78.2 (88.6)
Moderate + vigorous MVPA (min/wk)	141.8 (142.6)	187.3 (200.3)
Total physical activity (min/wk)	341.5 (260.2)	507.7 (333.1)
Body composition (BIA measure), mean (SD)		
Lean mass (kg)	41.6 (4.6)	43 (4.8)
Fat mass (kg)	23.7 (6.4)	22.5 (8)
Fat (%)	34.5 (4.9)	32.3 (6.7)
Body composition (DXA measure), mean (SD)		
Lean mass (kg)	40.3 (4.6)	41.5 (5.6)
Fat mass (kg)	26.5 (6.6)	25.2 (8.1)
Fat (%)	39.2 (4.9)	37.0 (6.9)
Vitamin D status, mean (SD)		
25-hydroxyvitamin D (μg/L)	34.1 (15.8)	34.7 (14.4)

Data are presented as mean and SD or as frequencies (*n*) and percentages (%).

Abbreviations: BIA, bioelectrical impedance analysis; DXA, dual-energy X-ray absorptiometry; MVPA, moderate and vigorous physical activity.

### HR-pQCT vBMD outcomes

Changes in HR-pQCT-derived bone parameters are shown in Table 2. Over 12 mo, both groups experienced modest declines in bone microarchitecture. For the primary outcome, distal tibia total vBMD, the probiotic group showed a greater reduction than the placebo group (between-group difference in change: −3.7 mg HA/cm^3^; 95% CI: −6.9, −0.4 mg HA/cm^3^; *P* = 0.027). This effect was not subject to multiplicity adjustment. At the tibia, cortical vBMD and cortical thickness declined in both groups, with greater reductions observed in the probiotic group; however, between-group differences did not reach significance after adjustment. At the radius, total vBMD declined in both groups, with a greater reduction in the probiotic group (between-group difference: −5.0 mg HA/cm^3^; 95% CI: −9.8, −0.1 mg HA/cm^3^; *P* = 0.044), although this did not remain significant after FDR adjustment. Other radial parameters showed similar directional changes but no significant between-group differences.

**TABLE 2:** Adjusted changes in tibia and radius high-resolution peripheral quantitative computed tomography markers from baseline to 12 mo in postmenopausal females receiving probiotic or placebo (linear mixed model analysis)

Outcome	Probiotic baseline (*n* = 58) adjusted mean (95% CI)	Probiotic adjusted change (95% CI)	Placebo baseline (*n* = 56) adjusted mean (95% CI)	Placebo adjusted change (95% CI)	Between-group difference in change (95% CI)	Raw *P* value	FDR-adjusted *P* value
Tibia total vBMD, mg HA/cm^3^	276.7 (264.1, 289.3)	−3.3 (−5.5, −1.0)	270.8 (258.2, 283.4)	0.4 (−1.9, 2.7)	−3.7 (−6.9, −0.4)	0.027	—1
Tibia cortical vBMD, mg HA/cm^3^	823.2 (807.6, 838.8)	−9.8 (−16.7, −2.9)	817.9 (802.3, 833.5)	−0.6 (−7.7, 6.6)	−9.3 (−19.2, 0.7)	0.067	0.101
Tibia cortical thickness, mm	0.981 (0.921, 1.042)	−0.019 (−0.029, −0.009)	0.954 (0.894, 1.015)	−0.004 (−0.015, 0.006)	−0.015 (−0.031, 0.000)	0.051	0.101
Tibia total BV/TV	0.136 (0.129, 0.144)	−0.003 (−0.004, −0.001)	0.135 (0.127, 0.142)	0.001 (−0.001, 0.002)	−0.003 (−0.006, −0.001)	0.010	0.070
Radius total vBMD, mg HA/cm^3^	301.3 (285.0, 317.6)	−9.3 (−12.7, −6.0)	299.4 (283.1, 315.7)	−4.4 (−7.8, −0.9)	−5.0 (−9.8, −0.1)	0.044	0.101
Radius cortical vBMD, mg HA/cm^3^	828.5 (812.4, 844.5)	−8.6 (−16.7, −0.5)	835.8 (819.8, 851.9)	−5.1 (−13.5, 3.2)	−3.5 (−15.1, 8.1)	0.554	0.554
Radius cortical thickness, mm	0.684 (0.640, 0.727)	−0.023 (−0.034, −0.012)	0.686 (0.643, 0.730)	−0.015 (−0.027, −0.004)	−0.008 (−0.023, 0.008)	0.314	0.366
Radius total BV/TV	0.129 (0.121, 0.137)	−0.004 (−0.006, −0.002)	0.126 (0.118, 0.134)	−0.002 (−0.004, 0.000)	−0.003 (−0.005, 0.000)	0.072	0.101

Values are adjusted estimated marginal means (95% CI) from linear mixed-effects models. Models included fixed effects for group, time, and group × time interaction, and were adjusted for baseline BMI, calcium intake, serum 25(OH)D, MVPA, and years since menopause.

The treatment effect is the group × time interaction, representing the adjusted between-group difference in change from baseline to 12 mo.

Sample sizes reflect the number of participants contributing data at each time point. Linear mixed models include all available observations under the missing-at-random assumption and do not require complete cases.

Raw *P* values correspond to the interaction term. FDR-adjusted *P* values were calculated using the Benjamini–Hochberg procedure across secondary HR-pQCT outcomes.

Abbreviations: BV/TV, bone volume to tissue volume ratio; CI, confidence interval; FDR, false discovery rate; HR-pQCT, high-resolution peripheral quantitative computed tomography; MVPA, moderate and vigorous physical activity; vBMD, volumetric bone mineral density; 25(OH)D, 25-hydroxyvitamin D.

1 Primary outcome; not adjusted for multiple comparisons.

### DXA aBMD outcomes

Changes in aBMD are presented in Table 3. Both groups experienced small declines in aBMD across all skeletal sites over 12 mo. At the lumbar spine, the probiotic group showed a greater reduction compared with the placebo group (between-group difference: −0.009 g/cm^2^; 95% CI: −0.017, 0.000 g/cm^2^; *P* = 0.042), although this did not remain significant after FDR adjustment.

**TABLE 3:** Adjusted changes in dual-energy x-ray absorptiometry areal bone mineral density from baseline to 12 mo in postmenopausal females receiving probiotic or placebo (linear mixed model analysis)

Outcome	Probiotic baseline (*n* = 58) adjusted mean (95% CI)	Probiotic adjusted change (95% CI)	Placebo baseline (*n* = 56) adjusted mean (95% CI)	Placebo adjusted change (95% CI)	Between-group difference in change (95% CI)	Raw *P* value	FDR-adjusted *P* value
Lumbar spine (L1–L4) aBMD, g/cm^2^	0.926 (0.896, 0.956)	−0.013 (−0.019, −0.007)	0.915 (0.885, 0.945)	−0.004 (−0.010, 0.002)	−0.009 (−0.017, 0.000)	0.042	0.056
Total hip aBMD, g/cm^2^	0.867 (0.841, 0.892)	−0.009 (−0.013, −0.004)	0.859 (0.834, 0.885)	−0.003 (−0.008, 0.001)	−0.005 (−0.012, 0.001)	0.108	0.108
Femoral neck aBMD, g/cm^2^	0.722 (0.695, 0.748)	−0.010 (−0.017, −0.003)	0.713 (0.686, 0.739)	−0.009 (−0.016, −0.002)	−0.001 (−0.011, 0.009)	0.867	0.867
Forearm aBMD, g/cm^2^	0.518 (0.505, 0.531)	−0.011 (−0.015, −0.008)	0.519 (0.506, 0.532)	−0.006 (−0.010, −0.003)	−0.005 (−0.010, 0.000)	0.040	0.056

Values are adjusted estimated marginal means (95% CI) from linear mixed-effects models. Models included fixed effects for group, time, and group × time interaction, and were adjusted for baseline BMI, calcium intake, serum 25(OH)D, MVPA, and years since menopause.

The treatment effect is the group × time interaction, representing the adjusted between-group difference in change from baseline to 12 mo.

Sample sizes reflect the number of participants contributing data at each time point. Linear mixed models include all available observations under the missing-at-random assumption and do not require complete cases.

Raw *P* values correspond to the interaction term. False discovery rate–adjusted *P* values were calculated using the Benjamini–Hochberg procedure across DXA aBMD outcomes.

Abbreviations: aBMD, areal bone mineral density; CI, confidence interval; DXA, dual-energy x-ray absorptiometry; FDR, false discovery rate; MVPA, moderate and vigorous physical activity; 25(OH)D, 25-hydroxyvitamin D.

At the forearm, bone loss was also greater in the probiotic group (between-group difference: −0.005 g/cm^2^; 95% CI: −0.010, 0.000 g/cm^2^; *P* = 0.040), with borderline significance after adjustment. No significant between-group differences were observed at the total hip or femoral neck.

### Calciotropic hormones and bone metabolism biomarkers

Changes in circulating biomarkers are presented in Table 4. There was no evidence of a treatment effect on calciotropic hormones or BTMs.

**TABLE 4:** Adjusted changes in calciotropic hormones and bone metabolism biomarkers from baseline to 12 mo in postmenopausal females receiving probiotic or placebo (linear mixed model analysis)

Outcome	Probiotic baseline (*n* = 56) adjusted mean (95% CI)	Probiotic adjusted change (95% CI)	Placebo baseline (*n* = 54) adjusted mean (95% CI)	Placebo adjusted change (95% CI)	Between-group difference in change (95% CI)	Raw *P* value	FDR-adjusted *P* value
Calciotropic hormones							
PTH, pg/mL	51.3 (46.0, 56.6)	1.0 (−3.0, 5.0)	54.6 (49.3, 59.8)	−1.8 (−6.0, 2.3)	2.9 (−2.9, 8.6)	0.327	0.837
Calcitonin, pg/mL	0.805 (0.561, 1.050)	−0.034 (−0.101, 0.033)	0.672 (0.428, 0.917)	−0.026 (−0.096, 0.044)	−0.008 (−0.105, 0.089)	0.866	0.946
Total vitamin D, μg/L	33.7 (29.6, 37.9)	3.1 (−2.0, 8.3)	34.6 (30.5, 38.8)	1.0 (−4.3, 6.3)	2.1 (−5.3, 9.5)	0.574	0.837
Vitamin K-dependent carboxylation biomarkers							
Undercarboxylated osteocalcin, ng/mL	2.85 (2.32, 3.38)	0.30 (−0.19, 0.80)	2.71 (2.19, 3.22)	0.09 (−0.41, 0.60)	0.21 (−0.50, 0.92)	0.558	0.837
Bone formation biomarkers							
PINP, ng/mL	70.0 (64.2, 75.8)	−2.1 (−6.5, 2.2)	67.3 (61.5, 73.0)	−1.9 (−6.4, 2.6)	−0.2 (−6.5, 6.1)	0.946	0.946
Osteocalcin, ng/mL	35.1 (32.2, 37.9)	−5.3 (−6.9, −3.7)	33.9 (31.1, 36.8)	−5.6 (−7.3, −3.9)	0.3 (−2.0, 2.7)	0.773	0.946
Bone resorption biomarkers							
CTX, ng/mL	0.536 (0.490, 0.582)	−0.013 (−0.049, 0.023)	0.487 (0.441, 0.533)	−0.001 (−0.038, 0.037)	−0.012 (−0.064, 0.040)	0.641	0.837
TRAP-5b, U/L	4.85 (4.34, 5.36)	0.33 (−0.14, 0.79)	4.99 (4.48, 5.50)	0.20 (−0.28, 0.68)	0.13 (−0.54, 0.79)	0.711	0.946
Osteoclast differentiation regulators							
RANKL, pmol/L	0.117 (0.106, 0.129)	0.178 (0.120, 0.235)	0.127 (0.116, 0.139)	0.212 (0.151, 0.273)	−0.035 (−0.119, 0.049)	0.415	0.837
OPG, pmol/L	4.92 (4.24, 5.60)	−0.86 (−1.69, −0.03)	4.76 (4.08, 5.44)	−0.27 (−1.14, 0.60)	−0.59 (−1.79, 0.62)	0.335	0.837

Values are adjusted estimated marginal means (95% CI) from linear mixed-effects models. Models included fixed effects for group, time, and group × time interaction, and were adjusted for baseline BMI, calcium intake, serum 25(OH)D (except when vitamin D was the dependent variable), MVPA, and years since menopause.

The treatment effect is the group × time interaction, representing the adjusted between-group difference in change from baseline to 12 mo.

Sample sizes reflect the number of participants contributing data at each time point. Linear mixed models include all available observations under the missing-at-random assumption and do not require complete cases.

Raw *P* values correspond to the interaction term. FDR-adjusted *P* values were calculated using the Benjamini–Hochberg procedure across calciotropic hormone and bone metabolism biomarker outcomes.

Abbreviations: CI, confidence interval; CTX, C-terminal telopeptide; FDR, false discovery rate; MVPA, moderate and vigorous physical activity; OPG, osteoprotegerin; PINP, procollagen type I N-terminal propeptide; PTH, parathyroid hormone; RANKL, receptor activator of nuclear factor κ-B ligand; TRAP-5b, tartrate-resistant acid phosphatase 5b; 25(OH)D, 25-hydroxyvitamin D.

Parathyroid hormone, calcitonin, and total vitamin D concentrations remained stable in both groups, with no significant between-group differences. Similarly, markers of bone formation (PINP and OC) and bone resorption (CTX and tartrate-resistant acid phosphatase 5b) showed small changes over time but did not differ between groups. Markers of osteoclast regulation (RANKL and osteoprotegerin) showed modest changes within groups; however, between-group differences were not significant. After adjustment for multiple comparisons, none of the biomarker outcomes demonstrated a statistically significant treatment effect.

### Renal, liver, and inflammatory biomarkers

Changes in renal, liver, and inflammatory biomarkers are presented in Table 5. Overall, there was no evidence of a differential treatment effect on these biomarkers over 12 mo. Renal function markers remained stable in both groups, with small increases in urea and minimal change in creatinine, and no significant group × time interaction. Similarly, liver enzymes showed modest, non-directional changes, with slight decreases in aspartate aminotransferase in the probiotic group and small increases in the placebo group, while alanine aminotransferase remained largely unchanged; however, none of these changes differed between groups. Markers of systemic inflammation also showed no significant treatment effects. Concentrations of TNF-α remained stable across groups, whereas high-sensitivity C-reactive protein increased slightly in the probiotic group and decreased in the placebo group. IL-6 increased modestly in both groups over time. Despite these within-group fluctuations, no significant between-group differences were observed, and none remained significant after adjustment for multiple comparisons.

**TABLE 5:** Adjusted changes in renal, liver, and inflammation biomarkers from baseline to 12 mo in postmenopausal females receiving probiotic or placebo (linear mixed model analysis)

Outcome	Probiotic baseline (*n* = 56) adjusted mean (95% CI)	Probiotic adjusted change (95% CI)	Placebo baseline (*n* = 54) adjusted mean (95% CI)	Placebo adjusted change (95% CI)	Between-group difference in change (95% CI)	Raw *P* value	FDR-adjusted *P* value
Renal function biomarkers							
Urea, mg/dL	29.0 (27.0, 31.0)	0.7 (−1.2, 2.5)	27.6 (25.7, 29.6)	0.3 (−2.2, 1.7)	0.4 (−2.3, 3.1)	0.779	0.998
Creatinine, mg/dL	0.728 (0.702, 0.755)	−0.005 (−0.023, 0.013)	0.708 (0.682, 0.735)	0.007 (−0.025, 0.012)	−0.012 (−0.038, 0.014)	0.375	0.875
Liver function enzymes							
AST, U/L	25.8 (23.3, 28.3)	−1.1 (−3.0, 0.9)	23.2 (20.7, 25.8)	1.6 (−3.6, 0.3)	−2.7 (−5.5, 0.1)	0.055	0.385
ALT, U/L	20.1 (17.7, 22.6)	−0.5 (−2.4, 1.5)	18.7 (16.2, 21.2)	1.8 (−3.8, 0.2)	−2.3 (−5.1, 0.5)	0.111	0.519
Inflammatory biomarkers							
TNF-α, pg/mL	6.05 (5.33, 6.76)	0.032 (−0.807, 0.742)	5.78 (5.06, 6.49)	0.031 (−0.839, 0.777)	0.002 (−1.118, 1.121)	0.998	0.998
IL-6, pg/mL	2.26 (1.82, 2.71)	0.571 (−1.050, 3.496)	1.74 (1.29, 2.18)	1.426 (−1.825, 2.855)	−0.855 (−2.711, 0.897)	0.998	0.998
hs-CRP, mg/L	2.48 (0.67, 4.29)	−1.22 (−3.50, 1.06)	2.66 (0.85, 4.47)	0.52 (−1.52, 2.56)	−1.74 (−5.02, 1.52)	0.293	0.875

Values are adjusted estimated marginal means (95% CI) from linear mixed-effects models. Models included fixed effects for group, time, and group × time interaction, and were adjusted for baseline BMI, calcium intake, serum 25(OH)D, MVPA, and years since menopause.

The treatment effect is the group × time interaction, representing the adjusted between-group difference in change from baseline to 12 mo.

Sample sizes reflect the number of participants contributing data at each time point. Linear mixed models include all available observations under the missing-at-random assumption and do not require complete cases.

Raw *P* values correspond to the interaction term. FDR-adjusted *P* values were calculated using the Benjamini–Hochberg procedure across renal, liver, and inflammation biomarker outcomes.

Abbreviations: ALT, alanine aminotransferase; AST, aspartate aminotransferase; CI, confidence interval; FDR, false discovery rate; hs-CRP, high-sensitivity C-reactive protein; MVPA, moderate and vigorous physical activity; 25(OH)D, 25-hydroxyvitamin D.

### Dietary intake indices

Changes in dietary intake are shown in Table 6. Over 12 mo, dietary patterns diverged modestly between groups, although most between-group differences in change were not significant. The probiotic group reported a reduction in energy intake over time, whereas the placebo group showed a slight increase. Calcium intake remained consistently lower in the probiotic group at both time points, with no significant group × time interaction. Within the probiotic group, small reductions were observed in fiber, magnesium, and phosphorus intake; however, between-group differences in change were not significant after adjustment for multiple comparisons. Macronutrient distribution and sodium intake remained broadly stable in both groups, with no evidence of a treatment effect.

**TABLE 6:** Adjusted changes in dietary intake from baseline to 12 mo in postmenopausal females receiving probiotic or placebo (linear mixed model analysis)

Outcome	Probiotic baseline (*n* = 58) adjusted mean (95% CI)	Probiotic adjusted change (95% CI)	Placebo baseline (*n* = 56) adjusted mean (95% CI)	Placebo adjusted change (95% CI)	Between-group difference in change (95% CI)	Raw *P* value	FDR-adjusted *P* value
Energy intake, kJ/d	8532.0 (8063.6, 9000.3)	−568.3 (−1305.7, 169.1)	8282.8 (7814.4, 8751.1)	160.9 (−609.7, 931.4)	−729.2 (−1795.7, 337.4)	0.178	0.521
Protein intake, % of kJ	17.9 (16.7, 19.2)	0.23 (−0.79, 1.25)	18.1 (16.8, 19.3)	0.69 (−0.38, 1.75)	−0.46 (−1.93, 1.02)	0.538	0.657
Protein intake, g/d	88.6 (83.0, 94.1)	−4.7 (−13.0, 3.6)	86.4 (80.8, 91.9)	5.7 (−3.0, 14.4)	−10.4 (−22.4, 1.7)	0.091	0.428
Fat intake, % of kJ	39.3 (37.2, 41.4)	−0.05 (−2.18, 2.29)	39.4 (37.3, 41.5)	0.67 (−1.66, 3.00)	−0.73 (−3.95, 2.50)	0.657	0.657
Fat intake, g/d	90.2 (82.8, 97.5)	−5.0 (−12.3, 2.4)	90.3 (82.9, 97.7)	0.55 (−7.13, 8.23)	−5.5 (−16.1, 5.1)	0.304	0.521
Carbohydrate intake, % of kJ	35.8 (33.7, 37.9)	−0.42 (−2.82, 1.99)	36.2 (34.0, 38.3)	−1.31 (−3.79, 1.17)	0.89 (−2.56, 4.34)	0.609	0.657
Carbohydrate intake, g/d	188.7 (173.2, 204.3)	−16.1 (−40.4, 8.2)	179.6 (164.0, 195.1)	3.3 (−19.9, 26.4)	−19.4 (−54.3, 15.6)	0.275	0.521
Dietary fiber intake, g/d	28.5 (25.9, 31.1)	−3.2 (−6.0, −0.44)	26.7 (24.1, 29.3)	−0.80 (−3.72, 2.11)	−2.4 (−6.4, 1.6)	0.236	0.521
Calcium intake, mg/d	823.4 (717.0, 929.9)	−49.5 (−142.6, 43.5)	1015.8 (909.4, 1122.3)	−2.7 (−99.6, 94.2)	−46.8 (−181.1, 87.5)	0.491	0.657
Magnesium intake, mg/d	387.8 (360.6, 415.0)	−36.3 (−62.3, −10.2)	374.4 (347.2, 401.6)	−5.3 (−32.7, 22.1)	−31.0 (−68.8, 6.8)	0.107	0.428
Phosphorus intake, mg/d	1531.7 (1438.8, 1624.5)	−153.1 (−276.3, −29.9)	1475.3 (1382.5, 1568.1)	1.8 (−126.0, 129.6)	−154.9 (−332.4, 22.6)	0.087	0.428
Sodium intake, mg/d	2287.2 (2069.8, 2504.6)	−102.6 (−402.1, 196.9)	2347.2 (2129.8, 2564.6)	−3.7 (−313.3, 305.9)	−98.9 (−529.7, 331.9)	0.650	0.657

Values are adjusted estimated marginal means (95% CI) from linear mixed-effects models. Models included fixed effects for group, time, and group × time interaction, and were adjusted for baseline BMI, calcium intake, serum 25(OH)D, MVPA, and years since menopause; the calcium intake model was adjusted for baseline BMI, serum 25(OH)D, walking time, and years since menopause, but not baseline calcium intake.

The treatment effect is the group × time interaction, representing the adjusted between-group difference in change from baseline to 12 mo. Sample sizes reflect participants contributing data at each time point; exact sample size may vary slightly across outcomes due to missing data. Linear mixed models include all available observations under the missing-at-random assumption and do not require complete cases. Raw *P* values correspond to the group × time interaction. FDR-adjusted *P* values were calculated using the Benjamini–Hochberg procedure across the 12 dietary intake outcomes.

Abbreviations: CI, confidence interval; FDR, false discovery rate; MVPA, moderate and vigorous physical activity; 25(OH)D, 25-hydroxyvitamin D.

## Discussion

This randomized, controlled trial found no evidence that 12 mo of lactobacilli combination supplementation improves bone health in early postmenopausal females. The primary analysis, based on linear mixed-effects models, showed a modest but statistically significant greater decline in distal tibia total vBMD in the probiotic group than in the placebo group. Although similar directional effects were observed at selected peripheral skeletal sites, these findings were small in magnitude and not consistently robust after adjustment for multiple comparisons. Importantly, no treatment effects were observed for BTMs, calciotropic hormones, or inflammatory biomarkers. The absence of concordant changes across biochemical and imaging outcomes suggests that the observed differences in bone density are unlikely to reflect a meaningful biological effect of the intervention. The magnitude of observed bone changes was small (∼1%–3%) and within the expected range of age-related bone loss, limiting clinical relevance. Furthermore, given the number of secondary outcomes assessed, the potential for chance findings remains despite adjustment for multiple comparisons.

Dietary intake patterns differed modestly between groups over the study period, with lower energy, protein, and calcium intake observed in the probiotic group at 12 mo, though these were non-significant. Although these differences were not the primary focus of the intervention, they may have contributed to the relatively greater bone loss observed in the probiotic group. However, residual confounding cannot be excluded, and these findings should be interpreted cautiously.

Systemic inflammation was low overall, limiting scope to observe anti-inflammatory effects. The blood concentrations of IL-6 rose modestly within the control group, whereas TNF-α remained stable, but between-group differences were not significant. Subgroup analyses indicated that females with higher baseline inflammation experienced a greater decrease in tibial vBMD when randomly assigned to probiotics, but these findings were inconsistent across sites and biomarkers. Because lactobacilli can produce menaquinones in fermented foods but are not major gut contributors of vitamin K_2_ in vivo [40], undercarboxylated OC was examined as a surrogate biomarker of vitamin K’s γ-carboxylation activity. However, no differences were detected between groups, suggesting that the tested strains were unlikely to exert a vitamin K_2_–mediated skeletal effect. Although DXA and HR-pQCT scans showed site-specific BMD changes, the changes in BTM levels did not differ between groups either. Such BMD–BTM dissociations have been noted in previous probiotic trials, especially in healthy human cohorts without high baseline remodeling [26,27,29,30,31,[34], [35], [36]]. These results reinforce that BTMs may be less sensitive for detecting modest microbiome-related effects in lower-risk populations over a 12-mo timeframe.

Bone loss after menopause is heterogeneous across compartments. In this trial, group differences were most apparent at trabecular-rich and peripheral sites, whereas cortical-dominant regions such as the hip and femoral neck showed no divergence. This aligns with prior reports of mixed, site-specific effects in probiotic trials [27,29,30,31,34]. Integrating multi-site assessments of HR-pQCT and DXA improves interpretability while underscoring the complexity of bone health. Although animal models consistently report probiotic benefits on bone through cytokine suppression, RANKL modulation, barrier effects, and possible vitamin K_2_ pathways [[21], [22], [23]], evidence from human clinical trials is inconsistent, attributed to strain heterogeneity, co-nutrient use, and population risk profiles [26,27,[29], [30], [31],[34], [35], [36]]. Exploratory subgroup analyses indicated that lower calcium intake, reduced walking time, overall physical activity, and higher baseline inflammation were associated with greater bone loss, particularly in the probiotic arm. These findings suggest that adequate nutrient status and regular activity remain prerequisites for skeletal preservation, irrespective of probiotic use. Furthermore, these findings align with a broader, equivocal evidence base where probiotic supplementation efficacy likely depends on specific strain selection, co-nutrient intake sufficiency, and population selection [29,35,36].

The host environment may account for the unfavorable response to probiotics observed in this trial. As previously reported, Nilsson et al. [30] demonstrated benefit in tibial total vBMD in the probiotic group compared to controls. A follow-up study [41] identified 10 “good responders” and 10 “poor responders” in the probiotic group (those with the highest or lowest changes in vBMD) to investigate the mechanisms underlying the effects of *Lactobacillus reuteri* by examining gut microbiome composition and serum metabolome. The authors reported significantly increased gene richness and improved inflammatory state in the good responders compared with the poor responders. This indicates that the host environment mediates the response to probiotic intervention. This observation may account for the less favorable response to probiotic intervention in the current study and may also explain the mixed findings of probiotic intervention in other clinical trials.

Strengths of this study include the randomized, double-blind design, the use of HR-pQCT for detailed skeletal assessment, and the application of linear mixed-effects models, which allowed inclusion of all available data and improved robustness of inference.

Limitations should also be acknowledged. First, dietary intake and physical activity were self-reported, introducing potential recall and reporting bias that may have obscured true behavioral differences between groups. Second, BTMs are inherently variable and may lack sensitivity to modest probiotic effects in relatively healthy populations over a 12-mo timeframe. Third, the magnitude of observed changes was small (∼1%‒3%) and within the expected range of age-related bone loss, limiting clinical relevance. Further, all BMD changes observed fell within the COV of the measurement techniques, further highlighting the need for caution in interpretation. By examining probiotics without concurrent calcium or vitamin D supplementation, the trial provided insight into probiotic effects in isolation; thus, the absence of nutrient co-supplementation may have limited the opportunity to observe a microbiome-mediated skeletal response. The generalizability of these findings is limited to postmenopausal females and should not be extrapolated to other age groups or to males. Finally, the trial by its design could not detect fracture outcomes, which are the most clinically relevant endpoints, and the 1-y duration may have been insufficient to observe cortical changes or longer-term skeletal adaptations.

In conclusion, daily supplementation with a lactobacillus combination resulted in significant between-group differences in certain BMD outcomes, with greater bone loss in the probiotic group observed at trabecular-rich skeletal sites (including tibial and radial vBMD, and spine and forearm aBMD). However, interpretation should remain cautious given the modest magnitude of change and potential influence of confounding factors. Ensuring adequate nutrition and maintaining regular physical activity remain the most reliable strategies for skeletal preservation in early postmenopausal females. Future public health research should explore barriers that limit the implementation of this low-cost, evidence-based approach to improving skeletal outcomes. Future trials should adopt multimodal approaches that combine probiotic supplementation with sufficient intake of key bone-related nutrients and structured weight-bearing or light physical activity (e.g., walking). Longer-term follow-up and mechanistic studies examining pathways, such as gut microbiota composition, short-chain fatty acid production, and vitamin K_2_ biosynthesis, are also needed to clarify potential benefits in specific subgroups. Probiotics may have a potential role in supporting skeletal health during early menopause; however, substantial additional research is needed to identify the appropriate target population, clarify gut microbiota profiles, and determine how specific strains may contribute within a broader lifestyle-based framework for osteoporosis prevention.

## Author contributions

The authors' responsibilities were as follows – GM, JRB, SR: designed the study; SR, TE, KY: conducted data collection; AV, HBFG: contributed to the biochemical analyses of bone metabolism biomarkers; AG-Z: conducted and analyzed the high-resolution peripheral quantitative computed tomography measurements; GM, SR: performed the statistical analyses; GM, SR, PD: drafted the manuscript; GM: had primary responsibility for the final content; and all authors: read and approved the final manuscript.

## Data availability

Data described in the manuscript, code book, and analytic code will be made available upon request pending application and approval.

## Declaration of generative AI and AI-assisted technologies in the writing process

The author(s) declare that no generative AI or AI-assisted technologies were used in the writing of this manuscript.

## Funding

The present study was funded by Probi AB. This research was supported by the Commonwealth through an Australian Government Research Training Program Scholarship (DOI: https://doi.org/10.82133/C42F-K220). The funders had no role in the study design, analysis, or writing of this manuscript.

## Conflict of interest

The authors report no conflicts of interest.
