# Supplementary material for: Effects of 12-month probiotic supplementation on bone mineral density and bone turnover markers in early postmenopausal females: A double-blind randomized controlled trial
Source: Am J Clin Nutr. 2026 Jun 8;124(2):101384. doi: 10.1016/j.ajcnut.2026.101384 (PMC13494076; doi:10.1016/j.ajcnut.2026.101384)
Supplement: Supplementary file 1 [file mmc1.docx]

**Effects of 12-Month Probiotic Supplementation on Bone Turnover Markers and Bone Mineral Density in Early Postmenopausal Females: A Double-Blind Randomized Controlled Trial**

**Stephanie Resciniti**

**Supplementary Table 1:** Description of assay type

| **Assays** | **Sample type** | **Type of assay** | **Name of manufacturer** | **Coefficients of variation** | **Limits of Detection (LOD)*** | **Outli-ers** |
| --- | --- | --- | --- | --- | --- | --- |
| UREA | Serum | Enzymatic | Abbott | 0.9% to 5.9% | 2 mg/dL | ND |
| CREA | Serum | Enzymatic -kinetic | Abbott | <5% | 0.06 mg/dL | ND |
| AST | Serum | Enzymatic | Abbott | <5% | 4 U/L | ND |
| ALT | Serum | Enzymatic | Abbott | <5% | 2 U/L | ND |
| Total Vitamin D | Serum | CLIA | Diasorin | <6% | 0.70 pg/mL | ND |
| Calcitonin | Serum | CLIA | Roche | <10% | 2 pg/mL | ND |
| PTH | EDTA plasma | CLIA | Abbott | <5% | 1 pg/mL | ND |
| OPG | Serum | ELISA | Biomedica | <10% | 0.07 pmol/L | ND |
| Rankl | Serum | ELISA | Biomedica | <10% | 0.01 pmol/L | ND |
| Osteocalcin | Serum | ELISA | Biomedica | 2.6% to 7.0% | 0.15 ng/mL | ND |
| CTx | Serum | CLIA | Roche | <5% | 0.01 ng/mL | ND |
| P1NP | Serum | CLIA | Roche | <4% | 5 ng/mL | ND |
| TRAP5 | Serum | ELISA | Quidel | <10% | 0.2 U/L | ND |
| hs-CRP | Serum | Immunotu-  rbidimetric | Abbott | <3% | 0.1 mg/L | ND |
| IL-6 | Serum | CLIA | Roche | <5% | 0.7 pg/mL | ND |
| TNF-α | Serum | CLIA | Snibe | <10% | 0.09 pg/mL | ND |
| ucOC | Serum | ELISA | Takara Bio | 4.4% to 9.9% | 0.25 ng/mL | ND |

Abbreviations: AST, aspartate aminotransferase; ALT, alanine aminotransferase; PTH, parathyroid hormone; OPG, osteoprotegerin; RANKL, receptor activator of nuclear factor-κB ligand, CTx, serum type I collagen cross-linked c-telopeptide, P1NP, amino-terminal procollagen 1 extension peptide, TRAP5, tartrate-resistant acid phosphatase isoform 5b; hs-CRP, high sensitivity C-reactive protein; IL-6, interleukin-6; TNF-α, tumour necrosis factor- α; ucOC, undercarboxylated osteocalcin; ND, Not detected.

*Only lower limits of detection were provided by manufacturers.
